# Somatization disorder among adolescents in southeast Nigeria: a neglected issue

**DOI:** 10.1186/s13033-017-0161-3

**Published:** 2017-09-21

**Authors:** A. R. C. Nwokocha, J. M. Chinawa, V. Onukwuli, A. Ubesie, Appolos Ndukuba, A. T. Chinawa, Elias Aniwada, Samuel Uwaezuoke

**Affiliations:** 1 Department of Pediatrics, College of Medicine, University of Nigeria, University of Nigeria/Teaching Hospital (UNTH), Ituku-Ozalla, Enugu State Nigeria; 2 Department of Psychological Medicine, University of Nigeria, University of Nigeria/Teaching Hospital (UNTH), Ituku-Ozalla, Enugu State Nigeria; 3 Department of Community Medicine, ESUT Teaching Hospital, Enugu, Enugu State Nigeria; 4 Department Community Medicine, College of Medicine, University of Nigeria, University of Nigeria/Teaching Hospital (UNTH), Ituku-Ozalla, Enugu State Nigeria; 5 Department of Pediatrics, UNTH, PMB 01129, Enugu, 400001 Enugu State Nigeria

**Keywords:** Psychosomatic disorder, Adolescents, Secondary schools, Nigeria

## Abstract

**Background:**

Adolescents do present with somatization disorder which is often neglected by pediatricians. This could have serious consequences if not curbed early.

**Objectives:**

This study is aimed at determining the pattern and types of Somatization disorder among adolescents attending secondary schools in south east Nigeria.

**Methods:**

Somatization disorder was investigated among 485 adolescents from mixed schools using a stratified random sampling of adolescents from four secondary schools in southeast Nigeria. The Enugu somatization scale was used to evaluate for presence of somatization in the participants. Statistical analysis was with statistical package for social sciences (SPPS) version 19 (Chicago IL).

**Results:**

A total of 485 adolescents aged 10–19 years were included in this study. The mean age of the respondents was 16.36 with standard deviation (SD) of 3.14 years. Two hundred and fifty-one (51.8%) had head features, 262 (54.0%) had body features, 303 (62.5%) had either head or body features while 210 (43.3%) had both head and body features. One hundred and thirty-four males (51.3%) compared to 117 females (52.2%) reported symptoms consistent with head symptoms (p = 0.038). One hundred and eleven males (42.5%) compared to 99 females (44.2) reported symptoms related to the head and body (p = 0.137) while 135 males (51.7%) compared to 127 females (56.7%) reported symptoms related to the body (p = 0.925). There were significant associations of age in categories with head, body, either head or body as well as both head and body features (all p value <0.001).

**Conclusions:**

Psychosomatic problems do exist and may be on the rise among adolescents.

**Electronic supplementary material:**

The online version of this article (doi:10.1186/s13033-017-0161-3) contains supplementary material, which is available to authorized users.

## Background

Somatization disorder (SD) as a psychiatric diagnosis became entrenched in the DSM-IV (2000) to reflect the condition hitherto known as Briquet’s syndrome which is a polysymptomatic condition that starts before the age of 30 years and extends for a long time [1]. It is characterized by pains, gastrointestinal, genitourinary and pseudo-neurological symptoms. In the ICD-10, somatization is defined as multiple, recurrent and frequently changing physical symptoms usually present for several years; (at least 2 years) before the patient is referred to a psychiatrist [2]. There are significant departures from the DSM-IV categorization which identified somatization disorder, hypochondriasis, pain disorder, and undifferentiated somatoform disorder. All these are now included under the heading of SD [2]. In addition, the symptoms need no longer to be medically unexplained but may or may not be associated with another medical condition. This implies that adolescents who had organic co- morbidities who were previously excluded under DSM-IV can now be included in the diagnosis of SD and be considered for appropriate treatment.

It has been generally believed that this disorder occurs predominantly in non-western and developing societies. However, there is growing evidence that suggests that somatic disorder is a hidden malaise that presents in large numbers all over the world, but is often under diagnosed [3]. This behavioral problem among adolescents is common in all cultural groups and societies, especially in the primary care [4]. With the burden of this problem in view, it is believed that it will give the pediatric adolescent specialist some hint that his patient, who may deny any psychic distress, is actually under some unbearable psychological states [5]. It is seen that when the adolescents is diagnosed with somatic problems, it makes the adolescent withdrawn from his peers and is often been stigmatized as been abnormal and psychotic. Thus the Nigerian adolescent cannot afford to even discuss this problem with his parents or teachers since he/she may be forced to cope with somatic distress for a long time [5–9].

Reported prevalence of somatization varies depending on the criteria. It has been noted that about 50% of adolescents who attend pediatric clinic will complain of medically unexplained symptoms with significant functional and emotional impairment. Such adolescents pose heavy burdens on the healthcare system through frequent utilization of health resources and hospitalizations, specialist consultations, unnecessary investigations, and treatments [10, 11]. In addition to the negative impact of the associated co-morbidities, family conflict and school absenteeism, failure to make accurate and timely diagnosis often results in multiple referrals, repeated unnecessary diagnostic tests, unjustified and potentially harmful treatments including medication trials and even surgeries, and the perpetuation of the belief of underlying organic illness [12].

Accurate diagnosis and treatment of adolescent somatic problem can make a great difference in patients’ life and in pediatrician or physician satisfaction. These treatment approaches involve a multidisciplinary approach which aims at targeting the adolescent/family’s understanding of the mind–body relationship and their acceptance of the bio-psycho-social formulation and treatment [13]. However, Ohaeri and Olatawura have observed the resilience of SD to physical methods of treatment [14].

Managing somatization disorder often poses challenges to the physician. The reasons would include ignorance of the condition [15]. As Ndukuba et al. [16] noted, there is a poor knowledge of conversion disorders by pediatricians in Nigeria and this could delay identification of children and adolescents with somatoform disorders. In addition, the drive to rule out organic disease, the fear of missing organic diseases and the poverty of skills in exploring psychological issues by most physicians could contribute to the difficulties faced by them in managing an adolescent presenting with this condition. Interestingly, Smith [17] had shown that the health care costs declined by 53% when primary care physicians treated their somatizing patients appropriately.

Considering that adolescents make up a significant proportion of the Nigerian population and that a recent study of adults with mental health disabilities documented that their problems reportedly started in early adolescence or around 14 years of age and given the serious negative impact of mental health problems, there is need to evaluate children and adolescents for somatization disorder, a very common mental health problem [18]. This is particularly so in south-east Nigeria where studies are scanty that have focused on childhood and adolescent somatic disorders.

This study is aimed at determining the types somatization disorder among adolescents attending secondary schools in South east Nigeria. It is hoped that this study will add to the knowledge of this disorder and will alert the pediatrician of its existence among children and adolescent age group.

## Methods

### Setting

The study was carried out among adolescents in four secondary schools in Enugu and Ebonyi States of south east Nigeria.

### Study design

A descriptive cross sectional study was used to identify the pattern and types of PSDs among adolescents attending secondary schools in South east Nigeria.

### Sampling

Four secondary schools were selected in Enugu and Ebonyi metropolis through stratified random sampling method. The schools were stratified using state and nature of school that is whether unisex or mixed schools. Two same gender schools and two co-educational schools were utilized. The schools selected were proportionately allocated number of participants. From a total 3654 adolescent students in all chosen schools, 485 adolescents were selected using random sampling.

### Data collection

A pretested self-administered Enugu somatization scale (ESS) developed by Ebigbo [6] was used for this study. The Enugu somatization scale is a culture specific screening scale that measures the culture-bound illness somatization. This instrument was developed bearing in mind the shortcomings or uncertainties that accompanies the use of western methods of assessment techniques and also to figure out the nature of psychological problem behind somatic symptoms as presented by Enugu, Nigerian patients. The ESS is a 65-item scale. The scale was found to distinguish patients from normals [6]. It has two sub-scales. The first is the HEAD subscale, captured by items 1–23, while the BODY subscale is captured by items 24–65. The ESS has a dichotomous response options which are YES and NO. It has been cross-validated with neurotic illness questionnaire (NIQ) in India where the ESS was found to correlate significantly with NIQ [7]. The ESS is a dichotomous response scale with YES and NO representing the dichotomies. A score of 1 is assigned to any Yes while a score of 0 is assigned to any NO response. With the exception of item 55 which is positively worded, all other items on the ESS are scored by the number of positive responses. Interpretation of score was based on the norm produced by the author. See Table 1.

**Table 1:** Norms for the Enugu summarization scale

	Students	
	Male	Female
Head		
Mean scores	3.58	4.12
Body		
Mean scores	7.22	7.73
Std. dev.	4.40	2.81

The questionnaires were completed by the students under strict examination conditions during class hours after explanation of the purpose of the study. Confidentiality was assured by informing the respondents not to write their names on the questionnaires. The class teachers were excluded from the class during the exercise to avoid their possible influence.

#### Data analysis

Statistical analysis was with statistical package for social sciences (SPPS) version 19 (Chicago IL). Chi square test was used to test for statistical association of age in categories as well as sex with presence or absence of psychosocial disorder. Binary logistic regression was used to control for confounding. All reported p values are 2-sided and values <0.05 were assumed as significant.

### Ethical consideration

Ethical approval for the study was obtained from the Health Research Ethics Committee of the University of Nigeria Teaching Hospital, Ituku-Ozalla, Enugu. Permission and approval were obtained from post primary education board of each state as well as from principals of each school studied. Assent was obtained from the students after a detailed explanation of the study objectives, procedures, risks and benefits. Informed consent was also obtained from the principals of the selected schools, who acted as the legal guardians of the students.

This article is aimed at determining the types and factors associated with PSDs among adolescents attending secondary schools in South east Nigeria. Subjects who gave consent were included in this study while those without consent and those with obvious psychiatric disorders were excluded.

## Results

### Demography

A total of 485 adolescents aged 10–19 years were included in this study. Their median age was 16 (1QR: 15–17) years. Two hundred and sixty-one (53.8%) were males. Table 2 shows the socio-demographic characteristics of respondents. The mean age of the respondents was 16.36 with standard deviation (SD) of 3.14 years. Majority were aged 15–19 years.

**Table 2:** Socio-demographic characteristics of respondents

Socio-demographic characteristics	Total (N) = 485	
	Frequency (n)	Percent (%)
Age in categories (years)		
10–14	64	13.2
15–19	421	86.8
Mean (SD)	16.36 (3.14)	
Sex		
Female	224	46.2
Male	261	53.8

### Prevalence of psychosocial disorder

Table 3 shows the distribution and pattern of Somatization disorder. Two hundred and fifty-one (51.8%) had head features, 262 (54.0%) had body features, 303 (62.5%) had either head or body features while 210 (43.3%) had both head and body features.

**Table 3:** Distribution and pattern of somatization disorder

Somatization disorder	Total (N) = 485	
	Frequency (n)	Percent (%)
Head		
Absence	234	48.2
Presence	251	51.8
Body		
Absence	223	46.0
Presence	262	54.0
Either head or body		
Absence	182	37.5
Presence	303	62.5
Both head and body		
Absence	275	56.7
Presence	210	43.3

### Gender and somatization disorder

One hundred and thirty-four males (51.3%) compared to 117 females (52.2%) reported symptoms consistent with head symptoms (p = 0.038). One hundred and eleven males (42.5%) compared to 99 females (44.2) reported symptoms related to the head and body (p = 0.137) while 135 males (51.7%) compared to 127 females (56.7%) reported symptoms related to the body (p = 0.925) as depicted in Table 4.

**Table 4:** Relationship between socio-demographic characteristics and somatization disorder

Socio-demographics	Total (N) = 485		Bivariate analysis χ^2^ (p value)	Multivariate analysis AOR (95% CI)
	Absence	Presence		
	Freq (%)	Freq (%)		
Head				
Age categories (years)				
10–14	49 (76.6)	15 (23.4)	23.674 (0.000)	1
15–19	185 (43.9)	236 (56.1)		4.2 (2.7–7.7)
Sex				
Female	107(47.8)	117(52.2)	0.038(0.845)	NA
Male	127(48.7)	134(51.3)		
Body				
Age categories (years)				
10–14	49 (76.6)	15 (23.4)	27.764 (0.000)	1
15–19	174 (41.3)	247 (58.7)		4.7 (2.5–8.6)
Sex				
Female	97 (43.3)	127 (56.7)	1.200 (0.273)	NA
Male	126 (48.3)	135 (51.7)		
Head or body				
Age categories (years)				
10–14	45 (70.3)	19 (29.7)	33.807 (0.000)	1
15–19	137 (32.5)	284 (67.5)		4.9 (2.8–8.8)
Sex				
Female	79 (35.3)	145 (64.7)	0.905 (0.341)	NA
Male	103 (39.5)	158 (60.5)		
Head and body				
Age categories (years)				
10–14	53 (82.8)	11 (17.2)	20.475 (0.000)	1
15–19	222 (52.7)	199 (47.3)		4.3 (2.2–8.5)
Sex				
Female	125 (55.8)	99 (44.2)	0.137 (0.712)	NA
Male	150 (57.5)	111 (42.5)		

### Age and somatization disorder

The median age among adolescents that had somatization disorder was 16 (13–19) years compared to 15 (range: 10–19) years among those without somatization disorder (U = .18555.0, p < 0.001). The median age of adolescents that reported head symptoms was 16 (range: 10–19) years compared to 15 (range: 10–19) among those without head symptoms (U = .21873.0, p = 0.002). Similarly, the median age among adolescents that reported body symptoms was 16 (range: 13–19) years compared to 15 (range: 10–19) among those without body symptoms (U = .21014.0, p < 0.001). The proportion of adolescents that reported somatization symptoms across the various ages is shown in Table 4.

Table 4 shows the relationship between socio-demographic characteristics and somatization disorder. There were significant associations of age in categories with head, body, either head or body as well as both head and body features (all p value <0.001). The respondents that were aged 15–19 years were; about 4 times (AOR 4.2; 95% CI 2.7–7.7) likely to have head features, about 5 times (AOR 4.7; 95% CI 2.5–8.6) likely to have body features, about 5 times (AOR 4.9; 95% CI 2.8–8.8) likely to have either head or body features and about 4 times (AOR 4.3; 95% CI 2.2–8.5) likely to have both head and body features than those aged 10–14 years.

## Discussion

The symptoms of psychosomatic disorders usually begin during adolescence or early adulthood and are characterized by many vague physical complaints. Any part of the body may be affected, although the symptoms and their frequencies vary. Common symptoms of this disorder among adolescents are headaches, nausea and vomiting, abdominal pain, diarrhea or constipation, fatigue, fainting, dizziness, sleeping problems, and nervousness. The most frequently seen symptoms among adolescents should be differentiated from psychosomatic disorders seen among adults [19].

Fifty-one point eight percent (51.8%) of these adolescents with this disorder reported symptoms related to the head while 54% reported symptoms involving the body and 62.5% had either head or body features while 43.3% had both head and body features. This high prevalence is similar to that obtained by Ibeziako et al. [11] who reported a prevalence of 50% but at variance with that of Gureje et al. [4] who found Nigerians to have less prevalence of somatization compared to Latin Americans. The prevalence obtained in this study is also higher than that of Arnold et al. [20] who reported a low prevalence of 17% in his study. Abdulbari et al. [21] also noted a prevalence rate of 23.9% among Qatari adults. Cultural and geographic construct as well as sample size, methodology and differences due to developmental age could also account for this variations in prevalence.

During the last decade, studies in several countries have shown that the proportion of adolescents with psychosomatic complaints (PSC) increased [5–9]. In Japan there is a considerable rise in the number of children presenting with psychosomatic disorders [22, 23]. The main symptoms in these children include headache, abdominal pain, and poor early rising. Children having two or more symptoms were reported to range about 20–30%. Pupils who come to school infirmaries with physical symptoms related to psychosomatic disorders have increased to more than 15% per week [24, 25].

It is not clear therefore whether the high prevalence found in this study reflects rising incidence of psychopathologies among the adolescent population. Could it be that these social and cultural changes are beginning to take a toll on the mental health of the adolescents?

The strength of this study is that it utilized an instrument developed locally, which considered the cultural issues specific to the region. One would therefore expect the respondents to understand the items of the instrument well and thus respond appropriately. On the other hand, it is likely that social desirability bias on the part of the respondents could have contributed to the high rates of endorsement seen in this study.

That nearly same proportion of participants that had symptoms referred to the head also had symptoms referred to the body could reflect the poly-symptomatic nature of the condition.

This study did not find any gender difference in the endorsement of symptoms except that males presented with head symptoms more than females. This is in agreement with other studies that have reported the obliteration of gender differences in most psychiatric disorders during adolescence [26].

This study finds that participants with symptoms were older than those without symptoms. The reason for this is not clear but considering that the condition is characterized by its chronic nature, it is likely that the number of adolescents with the condition would increase with increasing age.

The above discussion goes to portend the fact that a gamut of policy initiatives is needed to adequately provide for effective early mental health services for adolescents and their families. This policy must range from enhanced awareness of mental health issues and points of service access, development of effective techniques for identification and intervention, destigmatization of mental health problems, preparing pediatric psychologists for Collaborative roles in early childhood mental health services, funding for early childhood mental health service needs, family involvement in determination of service needs and policies and research aimed at broadening the range of effective diagnostic and intervention [27–29]. Apart from these policies, we also recommend rotations in child psychiatry for pediatric residents as an optional elective posting.

This study is limited by the fact that self-rated questionnaire was used. The response could be subject to social desirability bias. Moreover, the instrument being dichotomous makes it difficult to rate the severity of the symptoms. However, the strength of the study is in using a locally developed instrument that is culturally acceptable. This study contributed in highlighting a possible rise in prevalence of somatization disorder in this culture and the need to carry out more detailed study on this issue in the nearest future (Additional file 1).

## Conclusion

Psychosomatic problems do exist and may be on the rise among adolescents.

## Additional file

**Additional file 1.** Strobe statement checklist.

## Abbreviations

SD: somatization disorder
ESS: Enugu somatization scale
SPSS: statistical package for social sciences program
