# Supplementary material for: Somatization disorder among adolescents in southeast Nigeria: a neglected issue
Source: Int J Ment Health Syst. 2017 Sep 21;11:57. doi: 10.1186/s13033-017-0161-3 (PMC5609071; doi:10.1186/s13033-017-0161-3)
Supplement: Supplementary file 1 — Additional file 1. Strobe statement checklist. [file 13033_2017_161_MOESM1_ESM.doc]

STROBE Statement—Checklist of items that should be included in reports of ***cross-sectional studies***

|  | Item No | Recommendation |
| --- | --- | --- |
| **Title and abstract** | 1 | (*a*) Indicate the study’s design with a commonly used term in the title or the abstract  **This is indicated** |
|  |  | (*b*) Provide in the abstract an informative and balanced summary of what was done and what was found  **We have provided in the abstract an informative and balanced summary of what was done and what was found** |
| Introduction | | |
| Background/rationale | 2 | Explain the scientific background and rationale for the investigation being reported  **We have explained the scientific background and rationale for the investigation being reported** |
| Objectives | 3 | State specific objectives, including any prespecified hypotheses  **We have stated the specific objectives, including any prespecified hypotheses** |
| Methods | | |
| Study design | 4 | Present key elements of study design early in the paper  **We have presented key elements of study design early in the paper** |
| Setting | 5 | Describe the setting, locations, and relevant dates, including periods of recruitment, exposure, follow-up, and data collection  **We have described the setting, locations, and relevant dates, including periods of recruitment, exposure, follow-up, and data collection** |
| Participants | 6 | (*a*) Give the eligibility criteria, and the sources and methods of selection of participants  **We have given the eligibility criteria, and the sources and methods of selection of participants** |
| Variables | 7 | Clearly define all outcomes, exposures, predictors, potential confounders, and effect modifiers. Give diagnostic criteria, if applicable  **We have done this** |
| Data sources/ measurement | 8* | For each variable of interest, give sources of data and details of methods of assessment (measurement). Describe comparability of assessment methods if there is more than one group  **We have describe comparability of assessment methods if there is more than one group** |
| Bias | 9 | Describe any efforts to address potential sources of bias.  **This has been described** |
| Study size | 10 | Explain how the study size was arrived at. **We have explained the methods used in arriving at our sample size** |
| Quantitative variables | 11 | Explain how quantitative variables were handled in the analyses. If applicable, describe which groupings were chosen and why  **This is not applicable** |
| Statistical methods | 12 | (*a*) Describe all statistical methods, including those used to control for confounding  **We have described all statistical methods, including those used to control for confounding** |
|  |  | (*b*) Describe any methods used to examine subgroups and interactions.  **We have described methods used to examine subgroups and interactions**. |
|  |  | (*c*) Explain how missing data were addressed  **We have addressed this** |
|  |  | (*d*) If applicable, describe analytical methods taking account of sampling strategy  **We have describe analytical methods taking account of sampling strategy** |
|  |  | (*e*) Describe any sensitivity analyses  **We have describe any sensitivity analyses** |
| Results | | |
| Participants | 13* | (a) Report numbers of individuals at each stage of study—eg numbers potentially eligible, examined for eligibility, confirmed eligible, included in the study, completing follow-up, and analysed.**This has been addressed** |
|  |  | (b) Give reasons for non-participation at each stage.**We have given reasons** |
|  |  | (c) Consider use of a flow diagram. **Not applicable** |
| Descriptive data | 14* | (a) Give characteristics of study participants (eg demographic, clinical, social) and information on exposures and potential confounders.  **We have given characteristics of study participants (eg demographic, clinical, social) and information on exposures and potential confounders** |
|  |  | (b) Indicate number of participants with missing data for each variable of interest  **We have indicated number of participants with missing data for each variable of interest.** |
| Outcome data | 15* | Report numbers of outcome events or summary measures.  **This has been reported** |
| Main results | 16 | (*a*) Give unadjusted estimates and, if applicable, confounder-adjusted estimates and their precision (eg, 95% confidence interval). Make clear which confounders were adjusted for and why they were included.  **We have taken care of this request** |
|  |  | (*b*) Report category boundaries when continuous variables were categorized.  **We have reported category boundaries when continuous variables were categorized.** |
|  |  | (*c*) If relevant, consider translating estimates of relative risk into absolute risk for a meaningful time period. **Not applicable** |
| Other analyses | 17 | Report other analyses done—eg analyses of subgroups and interactions, and sensitivity analyses.  **We have reported the analyses of subgroups and interactions, and sensitivity analyses** |
| Discussion | | |
| Key results | 18 | Summarise key results with reference to study objectives.  **We have summarised key results with reference to study objectives** |
| Limitations | 19 | Discuss limitations of the study, taking into account sources of potential bias or imprecision. Discuss both direction and magnitude of any potential bias.  **We have discussed limitations of the study, taking into account sources of potential bias or imprecision.** |
| Interpretation | 20 | Give a cautious overall interpretation of results considering objectives, limitations, multiplicity of analyses, results from similar studies, and other relevant evidence.  **We have given a cautious overall interpretation of results considering objectives, limitations, multiplicity of analyses, results from similar studies, and other relevant evidence** |
| Generalisability | 21 | Discuss the generalisability (external validity) of the study results.  **We have discussed the generalisability (external validity) of the study results** |
| Other information | | |
| Funding | 22 | Give the source of funding and the role of the funders for the present study and, if applicable, for the original study on which the present article is based.  **We have given the source of funding and the role of the funders for the present study** |

*Give information separately for exposed and unexposed groups.

**Note:** An Explanation and Elaboration article discusses each checklist item and gives methodological background and published examples of transparent reporting. The STROBE checklist is best used in conjunction with this article (freely available on the Web sites of PLoS Medicine at http://www.plosmedicine.org/, Annals of Internal Medicine at http://www.annals.org/, and Epidemiology at http://www.epidem.com/). Information on the STROBE Initiative is available at www.strobe-statement.org.
